# Semi-automated protocol for purification of *Mycobacterium leprae* from tissues using the gentleMACS™ Octo Dissociator

**DOI:** 10.1016/j.mimet.2014.06.019

**Published:** 2014-10

**Authors:** Diana L. Williams, Linda B. Adams, Ramanuj Lahiri

**Affiliations:** Department of Health and Human Services, Health Resource Services Administration, Health Systems Bureau, National Hansen's Disease Programs, Laboratory Research Branch, SVM-LSU, RM 3517W, Skip Bertman Dr., Baton Rouge, LA 70803, USA

**Keywords:** Leprosy, gentleMACS Dissociator, Mouse footpad tissue, *Mycobacterium leprae*, Purification

## Abstract

*Mycobacterium leprae*, etiologic agent of leprosy, is propagated in athymic nude mouse footpads (FPs). The current purification protocol is tedious and physically demanding. A simpler, semi-automated protocol was developed using gentleMACS™ Octo Dissociator. The gentleMACS protocol provided a very effective means for purification of highly viable *M. leprae* from tissue.

## Highlights

• A gentleMACs protocol was developed for purification of *Mycobacterium leprae*.
• The current hand-held homogenization technique is tedious and physically demanding.
• Both protocols were compared using infected mouse footpad tissues.
• Both protocols produced highly viable, metabolically active *M. leprae* from host tissue.
• The gentleMACS protocol was less tedious, physically demanding and time-consuming.

*Mycobacterium leprae*, the causative agent of leprosy, cannot be grown axenically. The preferred system for cultivation of highly viable *M. leprae* for research purposes is the athymic nude mouse footpad (FP) model consisting of injecting *M. leprae* into each of the hind FP of nude mice and purification of bacteria from FP tissues 5–7 months postinfection (Truman and Krahenbuhl, 2001). The current protocol for purification of highly viable bacteria from this model requires extensive mincing of the tissue with scissors, the production of a tissue homogenate using a hand-held glass tissue grinder and differential centrifugation (Truman and Krahenbuhl, 2001). Although this technique produces highly purified, viable *M. leprae*, it is tedious, physically demanding and time consuming, especially when *M. leprae* is being harvested from large numbers of infected mice within a short time frame. Therefore, the objective of this study was to develop a simpler, semi-automated protocol for purification of *M. leprae* from infected FP tissues using a gentleMACS™ Dissociator.

These studies were performed under a scientific protocol reviewed and approved by the National Hansen's Disease Programs Institutional Animal Care and Use Committee (Assurance #A3032-01), and were conducted in accordance with all state and federal laws in adherence with PHS policy and as outlined in the Guide for the Care and Use of Laboratory Animals, Eighth Edition. Athymic *nu/nu* mice (Harlan Sprague–Dawley, Inc., Indianapolis, IN) were euthanized 5–7 months postinfection with *M. leprae* Thai-53. The hind legs were removed and decontaminated with Betadine for 20 min and rinsed in 4 washes of 70% ethanol. The skin was removed and the FP tissue was excised and weighed. For the purification of *M. leprae* from FP tissues using the gentleMACS protocol both feet were dissected into small pieces using a disposable scalpel. Tissue from a single mouse was added to a gentleMACS™ M tube (Miltenyi BioTec, www.gentleMACS.com) containing 10 ml RPMI-1640 (Life Technologies, Grand Island, NY) and 50 μg/ml ampicillin (Sigma-Aldrich) (RPMI/Amp). Tissues from 6 mice were simultaneously dissociated in a single run using the Protein 1 setting of a gentleMACS™ Octo Dissociator (Miltenyi BioTec) for 53 s and cooled on ice for 2 min, and the process was repeated twice. Each homogenate was transferred to a sterile 15 ml conical centrifuge tube and tissue debris was removed by centrifugation at 100 ×*g*, 2 min at 25 °C. Bacteria were pelleted at 10,000 ×*g*, 30 min at 4 °C, resuspended in RPMI/Amp + 10% FBS (Hyclone Laboratories, Logan, UT) and incubated at 37 °C for 1 h to remove potential microbial contaminants. Bacterial pellets were treated with 0.1 NaOH for 8 min to remove residual tissue debris and washed  3× in RPMI 1640 + 10% FBS Amp. For comparison, *M. leprae* were also purified from FP tissues using the standard hand-held homogenization technique (Truman and Krahenbuhl, 2001). Briefly, FP tissue from a single mouse was minced to a fine consistency for 3 min using a sterile pair of curved scissors. Tissue was transferred to a sterile 40 ml glass Wheaton Tenbroeck Tissue Grinder (Cat #08-414-13D, Fisher Scientific) and gently homogenized by hand on ice in 10 ml RPMI/Amp + 10% FBS for 5 min or until suspension looked milky. Bacteria were clarified, pelleted, incubated at 37 °C with Amp, and treated with NaOH as described above. Data from 6 mice per protocol were analyzed using two-tailed paired *t* test and compared by group using GraphPad software (www.graphpad.com/quickcalcs/ttest1.cfm) and considered significant at *p* value ≤ 0.05.

**Fig. 1:**
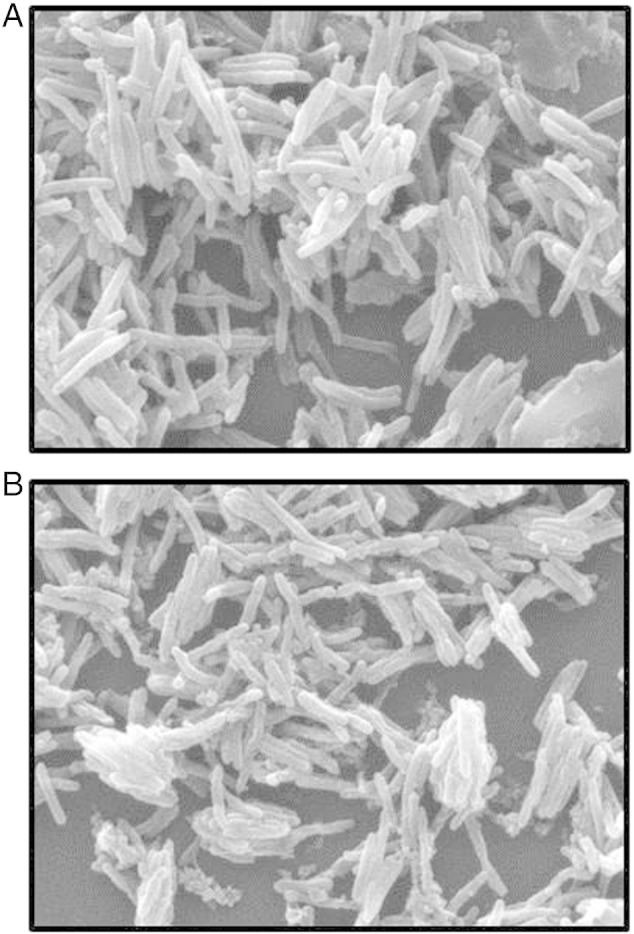
Scanning electron micrographs (10,000 × magnification) of purified *M. leprae* preparations from: A) hand-held homogenization protocol and B) gentleMACS protocol.

The hand-held homogenization protocol produced ~ 2-fold more *M. leprae* than the gentleMACS protocol according to acid-fast stain counts (*p*-value = 0.03) (Truman and Gillis, 2000) (Table 1). No significant differences were observed in the viability of *M. leprae* preparation using the LIVE/DEAD BacLight Bacterial Viability Kit (Molecular Probes) (Lahiri et al., 2005) and preparations exhibited similar levels of metabolic activity as determined using Buddemeyer radiorespirometry (Franzblau, 1988). Scanning electron microscopy using an FEI Quanta 200 scanning electron microscope showed that both bacterial preparations were relatively free of mouse cellular debris (Fig. 1).

**Table 1:** Comparison of gentleMACS vs hand-held homogenization protocols for purification of *M. leprae* from mouse footpad (FP) tissue.

Protocol	Bacterial yield^a^ AFB/mg FP tissue		% viability^b^		Radiorespirometry^c^ CPM/10^6^*M. leprae*	
	Mean	SD	Mean	SD	Mean	SD
gentleMACS Octo Dissociator	1.5 × 10^7^	0.59 × 10^7^^d^	82.5%	4.6%	3435	2056
Hand-held homogenization	3.4 × 10^7^	0.86 × 10^7^	84.5%	6.7%	4203	2592

a Bacterial yield = number of acid fast-stained bacterial counts/mg of FP (n = 6 mice/12 FP).

b % viability = LIVE/DEAD BacLight Bacterial Viability Kit (Molecular Probes).

c Radiorespirometry = Buddemeyer radiorespirometry (CPM)/10^6^*M. leprae* (7 d readings).

d Significance at *p* = 0.03 using two-tailed paired *t*-test.

A novel semi-automated procedure for the purification of *M. leprae* from infected tissues has been described. The gentleMACS protocol uses disposable scalpels and tissue dissociation tubes (M-tubes), eliminating the need for decontamination, cleaning and sterilizing of re-useable equipment such as scissors, forceps and glass homogenizers required for the hand-held homogenization protocol. Therefore, the preparation and clean-up time for a harvest is greatly minimized. The gentleMACS protocol FP homogenization step is automated while the hand-held method is very tedious and physically demanding, requiring 3 min of mincing with curved scissors prior to at least 5 min of gentle homogenization with a hand-held glass tissue grinder for each mouse. In addition, FP tissue from 8 mice can be homogenized in the gentleMACS Octo Dissociator in the same amount of time it takes to process the tissue from a single mouse with the hand-held homogenization method. Bacterial yields are ~ 2-fold lower with the gentleMACS protocol however viability and metabolic activity of *M. leprae* are comparable with both methods. We anticipate that this procedure will replace the current hand-held tissue homogenization protocol for purification of *M. leprae* from infected tissues for most research purposes. This will be especially beneficial when studies require large numbers of mice to be harvested in a short period of time, such as is common for vaccine or drug studies which can require 50–60 mice.

The authors wish to thank Cheryl Lewis, Felipe Sandoval, Nashone Ray, Baljit Randhawa, Vilma Marks and Gregory McCormick for their excellent technical assistance with this project. This project was supported by funding from the 10.13039/100000918New York Community Trust (Heiser Foundation for Leprosy and Tuberculosis Research #P12-000281), NIAID grant #IAA-2646, the American Leprosy Mission, and the 10.13039/100005561DHHS/10.13039/100000102HRSA/HSB/National Hansen's Disease Programs.
